# First-Principles Elucidation of the Surface Chemistry of the C_2_H_x_ (x = 0–6) Adsorbate Series on Fe(100)

**DOI:** 10.3390/molecules18043806

**Published:** 2013-03-26

**Authors:** Ashriti Govender, Daniel Curulla-Ferré, Manuel Pérez-Jigato, Hans Niemantsverdriet

**Affiliations:** 1 Sasol Technology R&D, PO Box 1, Sasolburg 1947, South Africa; 2 Gaz & Energies Nouvelles, Total S.A., Paris La Defense 6, France; 3 Physical Chemistry of Surfaces, Eindhoven University of Technology, PO Box 513, 5600 MB, Eindhoven, The Netherlands

**Keywords:** DFT, iron, ethane, ethylene, formation

## Abstract

*Ab initio* total-energy calculations of the elementary reaction steps leading to acetylene, ethylene and ethane formation and their decomposition on Fe(100) are described. Alongside the endothermicity of all the formation reactions, the crucial role played by adsorbed ethyl as main precursor towards both ethylene and ethane formation, characterises Fe(100) surface reactivity towards C_2_H_x_ (x = 0–6) hydrocarbon formation in the low coverage limit. A comprehensive scheme based on three viable mechanisms towards ethyl formation on Fe(100), including methyl/methylene coupling, methyl/methylidyne coupling followed by one hydrogenation and methyl/carbon coupling followed by two hydrogenations, is the main result of this article.

## 1. Introduction

Aiming at the remediation of the current world fuel shortage and spearheaded by heterogeneous catalysis, hydrocarbon production from natural gas, coal or biomass via synthesis gas (CO+H_2_) and the Fischer-Tropsch synthesis is at the heart of some of the main efforts currently taking place within the chemical energy industry. Furthermore, the investigation of fundamental adsorption and reaction properties of hydrocarbons on metal single crystal surfaces under ultra-high-vacuum conditions has become part of the foundation as well as a significant avenue towards the progress of modern surface science and catalysis [1].

The Fischer-Tropsch synthesis (FTS) is a versatile process that can be tuned to alkanes in the diesel and wax range using high-pressure–low-temperature conditions and cobalt or iron catalysts, or to shorter hydrocarbons, including small olefins, using high-pressure–high-temperature conditions and iron catalysts. Understanding the fundamental surface chemistry of paraffinic and olefinic hydrocarbons on the metals involved is an essential requirement for obtaining molecular level insight in (details of) the FTS mechanism.

There are typically three popular FTS mechanisms [2], although much debate on the subject still lingers on. The most accepted is the *carbide mechanism* which entails CO adsorption and dissociation towards adsorbed carbon and oxygen atoms, as well as successive hydrogenation of surface carbon atoms towards CH_x_ fragments, and insertion of CH_x_ monomers into the metal-carbon bond of an adsorbed alkyl chain. With methyl as chain initiator, methylene is considered the frame-building monomer. Chain termination takes place via the dehydrogenation of the resulting alkyl adsorbates towards an α -olefin, or the incorporation of an OH group to form n-alcohols.

The *enol mechanism* involves the partial hydrogenation of adsorbed CO to an enol, an oxygen-containing surface species, its chain growth occurring via the condensation of pairs of -CHOH species, via water elimination towards adsorbed -CHROH. Termination steps lead to oxygenates and α-olefins, with alkanes forming in a secondary step.

The *CO insertion mechanism* proceeds via the insertion of adsorbed CO into the metal-alkyl bond, leading to a surface acyl species, its chain termination taking place by either hydrogenation or dehydrogenation towards olefins, paraffins, aldehydes or alcohols. 

Understanding the composition of the resulting complex mixture in FTS, has become a crucial information source for its mechanism, the Anderson-Schulz-Flory product distribution comprising the main description in terms of chain-length [3], on the basis of a stepwise full-polymerisation model reaction. There exist many FTS mechanistic studies [4,5,6,7,8,9,10,11,12,13,14,15,16,17,18,19,20,21,22,23,24] that have been reported in the literature, mainly on Fe, Ni, Co and Ru. Some of them involve the investigation of relevant CH_x_ species and include the possibility of two different active sites driving independent reactions. Considering the large number of adsorbed species present during FTS on the catalyst surface, it does seem likely that more than one mechanism is at play.

Several *ab initio* studies of the chain-growth process of linear hydrocarbons on metal surfaces appear in the literature, probably the most detailed being the *carbide mechanism* based on reports by van Santen and co-workers [25,26,27], as well as those by Hu and co-workers [28,29,30,31]. Studies addressing higher level of molecular complexity for chain growth (including CO insertion, CHO insertion and synchronic C-C coupling/CO dissociation) have appeared in the literature [32,33,34], some on the iron carbide surfaces [35,36].

Lo and Ziegler [37] recently reported two-carbon adsorbate formation on Fe(100) from *ab initio* calculations. They found that most two-carbon species favour adsorption at the hollow site and that ethylene prefers to be π-bonded on Fe(100). On the other hand, they identify adsorbed ethyl on the bridge site, on Fe(100), and ethane in the physisorbed state, on the same surface. In that study, ethynyl and vinylidene are the most thermodynamically stable (ethylidyne is next in stability). Lee *et al.* [38] studied the decomposition of acetylene on Fe(001) from planewave-pseudopotential calculations. They concluded that the decomposition of acetylene by C-H bond breakage: CHCH → CCH → C+CH → C+C was just as likely as C-C bond breakage: CHCH → CH+CH → C+CH → C+C. The full dissociation of acetylene into atomic carbon is energetically favourable and exothermic. Anderson and Mehandru [39,40] calculated the adsorption of acetylene on Fe(100), (110) and (111) clusters, using the atom superposition and electron delocalisation molecular orbital theory (ASED-MO) method. They found that the high coordination hollow sites are favoured on (100) and (110) surfaces and the bridging site is favoured on Fe(111).

Cheng *et al.* [41] report the promotion effect of transition metal adsorbed atoms on ethylene chemisorption on Co(0001), from linear-combination of atomic-orbitals *ab-initio* calculations, and emphasize the role of Pd and Cu in decreasing ethylene adsorption energy, with the ensuing improvement in α-olefin selectivity. Xu *et al.* [42] present photoemission evidence for the coverage dependent ethylene decomposition on Co(0001) via acetylene, and identify several adsorbed carbon species, including dicarbon. The latter is identified as a stable species on fcc-Co(111), from *ab-initio* calculations, by Ciobîca and co-workers [43,44,45,46].

Several *ab initio* reports on the adsorption of ethylene on Ni(100) [47,48], Ni(110) [49,50,51,52] and Ni(111) [53] are known from the literature. A comparative investigation of ethylene dissociation on both Ni(111) and Ni(211) by Vang *et al.* [53] concludes that the C-C dissociation barrier is lowered at steps much more than the dehydrogenation barrier does.

Our group has published on important steps in the formation of hydrocarbons on the (100) surface of iron, being the direct dissociation of CO [54,55], the H-assisted dissociation of CO [56], the subsequent formation of water from adsorbed oxygen and hydrogen [57], and the formation of CH4 from adsorbed carbon and hydrogen [58]. This article completes the series with the ab-initio characterisation of the surface reactivity of Fe(100) towards C2 hydrocarbon formation in the low coverage regime. We believe our mechanism to provide understanding towards the process of linear hydrocarbon formation conditions in general.

A short justification for using the Fe(100) surface as a substrate for the reactions is in order. It is well known that metallic iron is unstable under FTS conditions and that iron is converted to carbides during early stages of the reaction [59,60]. We see the studies on Fe(100) as a simplified reference case for those on the much more complicated iron carbide surfaces, among which several have relatively similar surface free energy and stability [61]. We already published a preliminary study on the methane formation on Fe_5_C_2_ [62] and further investigations are in progress. 

This paper comprises the Results and Discussion inSection 2, the computational methodology as largely covered by references [63,64,65,66,67,68,69,70] in Section 3 and the conclusions in Section 4. Within Section 2, we first discuss the adsorption behaviour of C_2_H_x_ (x = 0–6) species, followed by co-adsorption configurations and minimum energy pathways. After determining the viability of the elementary steps in terms of barrier, we present the potential energy surfaces for acetylene, ethylene and ethane. After identifying three likely individual mechanisms, we bring these together into a comprehensive scheme describing a selection of FT reactions.

## 2. Results and Discussion

The stability of adsorbed hydrocarbon molecules and intermediates is hereby considered by means of two different thermodynamic properties, adsorption energy and chemical potential. Whereas the absolute stability scale is given by the adsorption energies of the surface species, the relative stability scale of chemical potentials provides enthalpies under a constant supply of adsorbed atomic hydrogen atoms, therefore allowing the comparison of the energetics of two adsorbed moieties that possess a different number of hydrogen atoms in their chemical formulas, as required by the study of chemical reactions.

The build-up of chemical potential profiles for the reactions of formation of adsorbed acetylene, ethylene and ethane involves the full study of all possible intermediates, starting from adsorbed atomic hydrogen, carbon and C_x_H_y_ moieties, and systematically progressing by chemical reaction towards the three original adsorbed molecules. In order to scan the ensemble of involved intermediates, the following reactions are studied; carbon-carbon coupling, hydrogenations, isomerisations and dehydrogenations. The methanation reaction has already been reported [58], and is not to be considered any further. 

### 2.1. Acetylene, Ethylene, Ethane and Intermediates: Stable Adsorption States

In order to identify all minima for acetylene, ethylene and ethane as well as all intermediates in between, geometry optimisations and (vibrational) normal-mode analysis are carried out. A careful sampling of optimal geometry enthalpy lets us choose the lowest energy configurations, and the normal-mode analysis lets us identify true minima by ensuring that all vibrational frequencies are real. Table 1 describes the lowest energy configuration for all the adsorbed species considered in terms of geometries, adsorption energies and vibrational frequencies, including *dicarbon*, *ethynyl*, *vinylidene*, *acetylene*, *vinyl*, *ethylidyne*, *ethylidene*, *ethylene*, *ethyl* and *ethane*.

The complete set of optimal adsorbate geometries and vibrational frequency data is available in a PhD thesis [70]. C_2_ adsorbates with no hydrogen atoms on the carbon bound to the metal, - C_α_-, tend to have the highest adsorption energy, and as the α-carbon gets more and more hydrogenated (until the α-carbon is fully hydrogenated), the adsorption bond becomes destabilised. The most stable moieties are dicarbon (adsorption energy −8.78 eV), ethynyl (adsorption energy −6.28 eV), and ethylidyne (adsorption energy −6.26 eV), although the dicarbon formation reaction is shown below not to be viable in the low coverage regime hereby considered. On the other hand, the molecule with the lowest adsorption energy is ethane, which is physisorbed, and will probably leave the surface as soon as it forms. 

The species involved in acetylene, ethylene and ethane formation (C_2_H_x_, x = 0–6), adsorb on fourfold hollow sites, except for ethyl that sits on the bridge site, and ethane, which does not exhibit any preference for any particular site on Fe(100). Whereas dicarbon, acetylene, ethylene and ethane adopt a flat configuration on Fe(100) in their most stable adsorption geometry, ethylidyne is the only species that stands completely upright on the surface. Ethynyl, vinyl, ethyl, vinylidene and ethylidene all exhibit a tilted geometry, with vinyl adopting an asymmetric (with the two β-hydrogens skewed) configuration on Fe(100), see Table 1.

The resulting dicarbon geometry is at variance to a previous *ab-initio* study by Lo and Ziegler [37] that suggests tilted structures. On the other hand the ethynyl (CCH) geometry is in agreement with that reported by the same authors, and the hereby reported vibrational frequencies for CCH compare well with experimental values from HREELS [71]. Furthermore, the vibrational frequencies hereby computed for acetylene are comparable to reported experimental frequencies from RAIRS [72]. Our optimal structure for vinyl on Fe(100) differs from that reported by Lo and Ziegler, but the ethylidene geometry is in agreement with theirs. The quad–σ-bonded configuration (hollow site) of acetylene does not agree with Lo and Ziegler, who report a π-bonded ethylene geometry (ontop) as the most stable. However, our result is in agreement with the experimental geometry reported by Hung and Bernasek [72]. All structures in Table 1 are characterised by real vibrational frequencies, except for the physisorbed ethane, and a low frequency of 36*i* cm^−1^ for ethylidyne. We nevertheless accept the ethylidyne in the hollow geometry as the most stable structure as a) it exhibits the strongest adsorption of all geometries considered [70] and b) adsorbate–surface coupling is not considered in the frequency calculations, which makes very low frequencies somewhat doubtful. 

**Table 1:** Lowest energy configuration for all intermediates in acetylene, ethylene and ethane formation and decomposition reactions, along with heats of adsorption, specific structural parameters and vibrational frequencies.

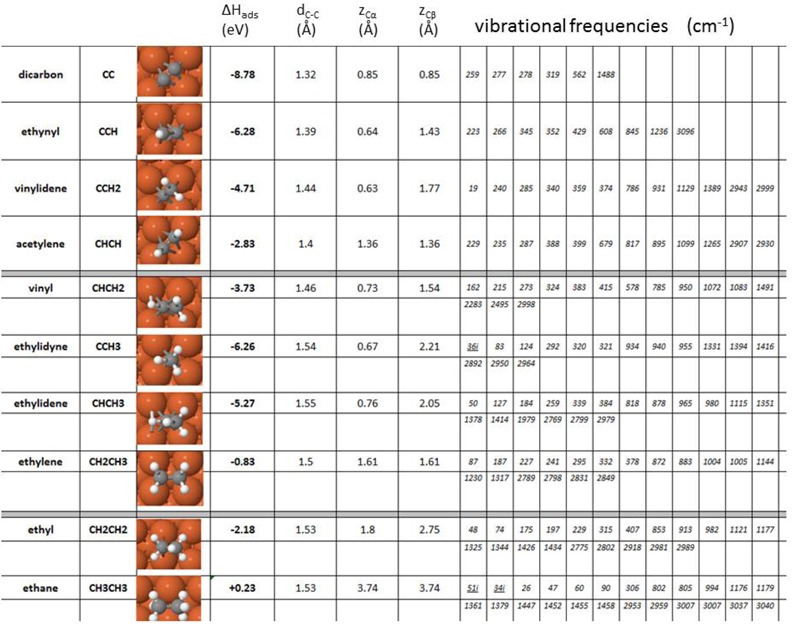

### 2.2. Elementary Reaction Steps Connecting Reaction Intermediates and Molecules: Co-Adsorption Configurations and Minimum-Energy Paths

Geometry optimisations for different co-adsorption systems, as required by the chemical reactions hereby investigated, are followed by vibrational normal-mode analysis. After identifying minima and transition states, the selection of initial state for reactions is followed by the computation of minimum-energy paths, in order to characterise elementary reactions. Isomerisation steps do not require the study of co-adsorption systems, usually implying adsorbates are in their ground state geometry.

**Figure 1:**
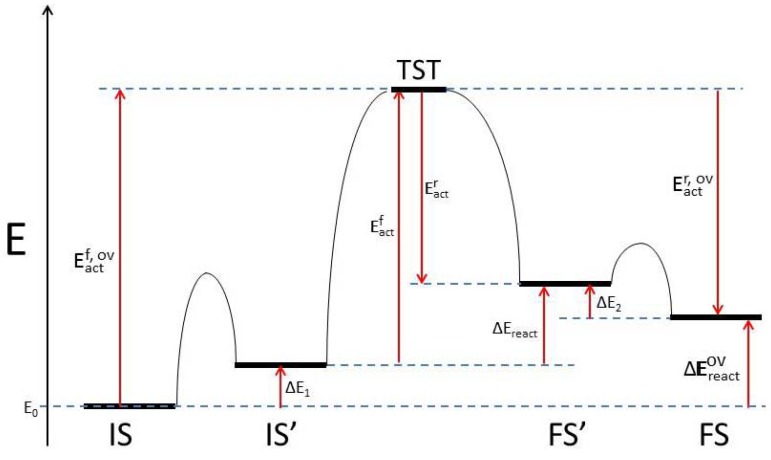
Schematic enthalpy diagram for a multi-step chemical reaction, as per the nomenclature of Table 2.

Table 2 describes the elementary steps linking all intermediates appearing during acetylene, ethylene and ethane formation and decomposition, in terms of the geometry of initial (IS), transition (TST) and final (FS) state, as well as forward and reverse activation energies and reaction energies. Pre-reaction and/or post-reaction steps are described in Table 2 whenever relevant (see the discussion section). Figure 1 illustrates the nomenclature used in Table 2. In case more than one pre-step is involved, we include the total change in energy before the main activation barrier as ΔE_1_. Table 2 represents a selection of elementary reactions starting from either the most stable or the most likely configurations. The complete set of co-adsorption geometries and minimum-energy path data is available [70].

### 2.3. Surface Reaction Mechanisms

In order to arrive at mechanistic descriptions of the formation or decomposition of C_2_ hydrocarbons, the viability of the elementary reaction steps is further investigated by means of the individual barrier and total barrier concept. All data in Subsection 2.2 are categorised below in terms of chemical potentials for all the species (including co-adsorbates) involved in the reaction sequence up to the formation of ethane, which provide a relative stability scale. A selection is then carried out on the basis of reaction barriers (either apparent or total), which leads to our mechanistic conclusion for the reaction of formation of C_2_H_x_ hydrocarbons.

Two separate approaches can be followed for interpreting the viability of the computed elementary reaction steps, when more than one stage is present. In the first, the concept of *individual reaction barrier/rate determining step* (RDS) is applied, and the main assumption in our energy barrier analysis is that the activation energy of the main-reaction has the highest value of all sequential steps (Figure 1). Provided a pre-reaction or post-reaction step had a higher activation energy than the main reaction, the new highest energy barrier value would be taken as the determining step. However, virtually all cases to be described in Table 2 comply with the situation sketched in Figure 1. Another assumption in this first analysis is that any pre-reaction or post-reaction stages accompanying the main reaction are all uncoupled from each other, in contrast to reactions that take place via a transient intermediate or to non-equilibrium multi-stage processes. The second analysis approach involves the complete picture provided by *the total/apparent reaction barrier*. The indicator employed now for the viability of the surface reactions under scrutiny is *the maximum enthalpy deviation*, the difference between the highest enthalpy value for all states (usually a transition state) and the lowest enthalpy value of the initial state (usually the ground state configuration for the initial state). Our work is limited by the fact that not all barriers are computed for pre- and post-reaction steps. Table 2 includes both, the rate determining step barrier corresponding to the individual thermal reaction rate (*individual* or RDS barrier); and the maximum enthalpy deviation for the total thermal reaction rate method (*total* or *apparent* barrier).

**Table 2:** Elementary steps for C_2_H_x_ (x = 0–6) formation, including (**a**) acetylene formation steps, and (**b**) ethylene and ethane formation steps, along with energy parameters as defined in Figure 1.

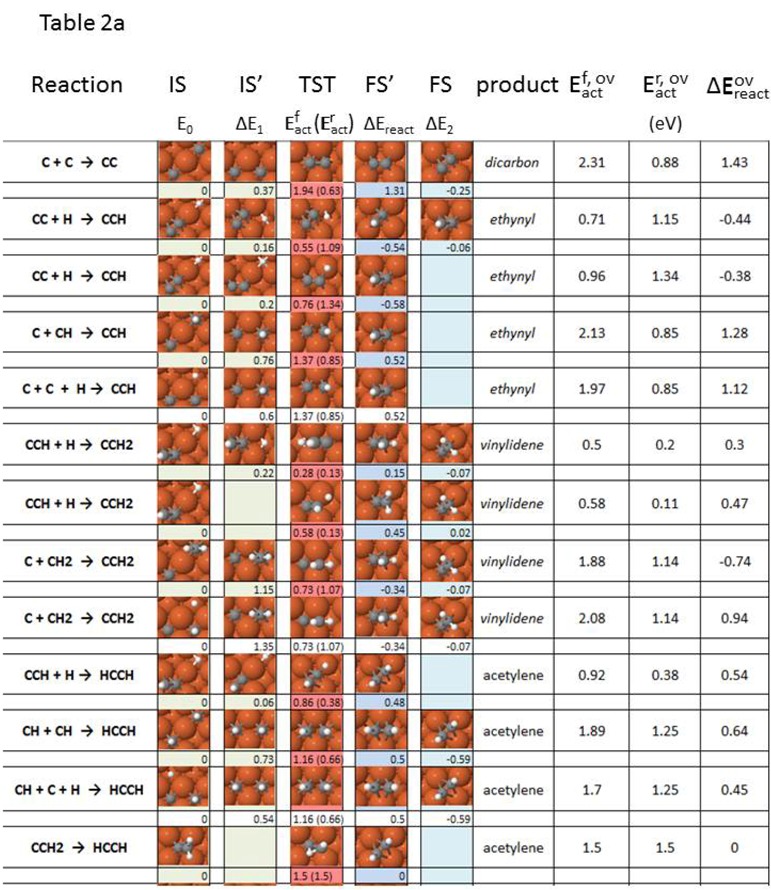
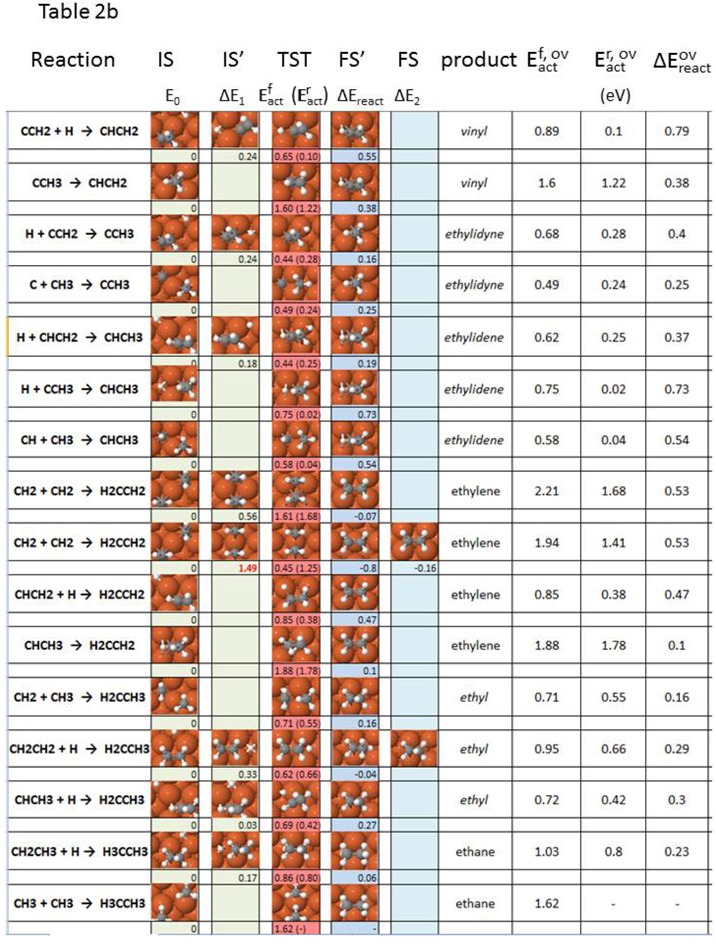

### 2.4. Chemical Potential Profiles for Acetylene, Ethylene and Ethane Formation

**Figure 2:**
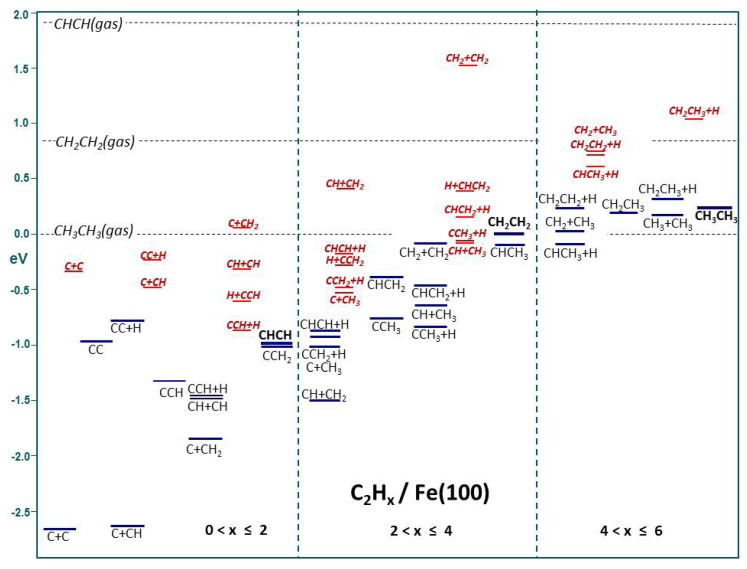
Compilation of chemical potential profiles of C_2_H_x_ for x = 0–6; transition states are indicated in red. The *x*-axis shows the systematic H-addition while the *y*-axis expresses energy in electronvolts (eV).

The information presented in Table 2 describes a complete series of elementary reaction steps connecting the adsorbed intermediates that appear on the Fe(100) surface within the process of formation of the three adsorbed hydrocarbons containing two carbon atoms; acetylene, ethylene and ethane, the steps connecting each of the formed hydrocarbons with the intermediates of the other hydrocarbons being included. The complete uninterrupted sequence of reaction steps up to the formation of adsorbed ethane provides a full description of the system reactivity, both in terms of C-H and C-C couplings. Figure 2 shows the total chemical potential profile for all adsorbed intermediates and molecules. The common scale used for Figure 2 corresponds to the ethane gas at zero energy and corresponding 2C+6H stoichiometry. On the other hand, the x-axis in Figure 2 follows a logical ordered sequence of adsorbed species complexity, starting from dicarbon and two co-adsorbed carbon atoms, and progressing step-wise via acetylene and ethylene/ethylidene up to ethane.

All elementary reaction steps connecting *dicarbon*, *ethynyl*, *vinylidene and acetylene* to acetylene formation and decomposition comprise the first section of Figure 2, including the steps linking. The sequence commences with C+C coupling to form dicarbon. The chemical potential for the reacting configuration of the co-adsorbed C/C system (initial-state) provides the lowest value in Figure 2 (neither the pre-reaction nor the post-reaction steps described in Table 2 for this reaction are included).

The chemical potential of ethynyl, CCH, is even more negative than that for dicarbon, as seen from Figure 2, and it is only overcome by the more negative values of some co-adsorption systems, viz C+C, C+CH, CH+CH, CCH+H. Our relative stability of ethynyl is in agreement with the high stability found experimentally for ethynyl being abundant at low hydrogen gas pressures on Fe(100), by Bernasek and co-workers [71,72]. Vinylidene and acetylene have similar chemical potentials, between those for dicarbon and ethynyl. The least negative of the series being that for dicarbon, the most negative that for ethynyl. Still, there are two co-adsorption systems with even less negative chemical potential values than all four intermediates/molecules (they are the least negative of all IS and FS in Figure 2), viz CC+H and C+CH_2_, these being very similar to each other.

The ethylene formation/decomposition sequence follows in Figure 2. The specific adsorbed species involved in this sequence of elementary steps include *vinyl*, *ethylidyne*, *ethylidene and ethylene*. The C+CH_3_ co-adsorbate in its ground state geometry, along with the acetylene and vinylidene adsorbates within the second relative scale, have similar chemical potentials, the most negative of all chemical potential values for C_2_H_x_ (2 < x ≤ 4). The least stable intermediate/molecule is ethylene, a preferred product in experimental FTS on iron catalysts, and the second least stable is ethylidene. The next intermediate in the relative stability scale is vinyl, and a more negative value corresponds to ethylidyne. The involved co-adsorption systems follow the chemical potential sequence C+CH_3_ < CCH_2_+H < CH+CH_2_ < CHCH+H < CCH_3_+H < CH_2_+CH_2_ < CH+CH_3_ < H+CHCH_2_ < CHCH_2_+H. Similarly to what happens for acetylene formation, and in the methanation chemical potential profile on Fe(100) reported by Govender *et al.* [58], all forward elementary reaction steps leading to ethylene formation, are endothermic.

The chemical potential profile describing the ethane formation reaction rounds off Figure 2. The two intermediates formed in this range of reactions are *ethyl* and *ethane*. The most stable adsorbed species now, which in turn have negative chemical potentials, are ethylidene and the ethylidene-hydrogen co-adsorption system, with similar chemical potentials. They are followed by ethylene, and then the CH_2_+CH_3_ co-adsorption system. The remaining chemical potential values for adsorbed intermediates-molecules as well as for co-adsorbates follow the sequence *CH*_2_*CH*_3_+*H* < *CH*_2_*CH*_3_ < *CH*_3_*CH*_3_ < *CH*_2_*CH*_2_+*H* < *CH*_3_+*CH*_3_. Adsorbed ethane appears at chemical potential very close to that for CH_2_CH_2_+H.

### 2.5. Selection of Three Viable Reaction Mechanisms

Considering that high temperature Fischer-Tropsch synthesis is carried out up to 625 K, and that this temperature corresponds to an overall kinetic barrier in the order of 1.5 eV, we will not consider reactions with barriers well above this value, and particularly not in cases where alternatives at more favourable barriers are available. 

**Figure 3:**
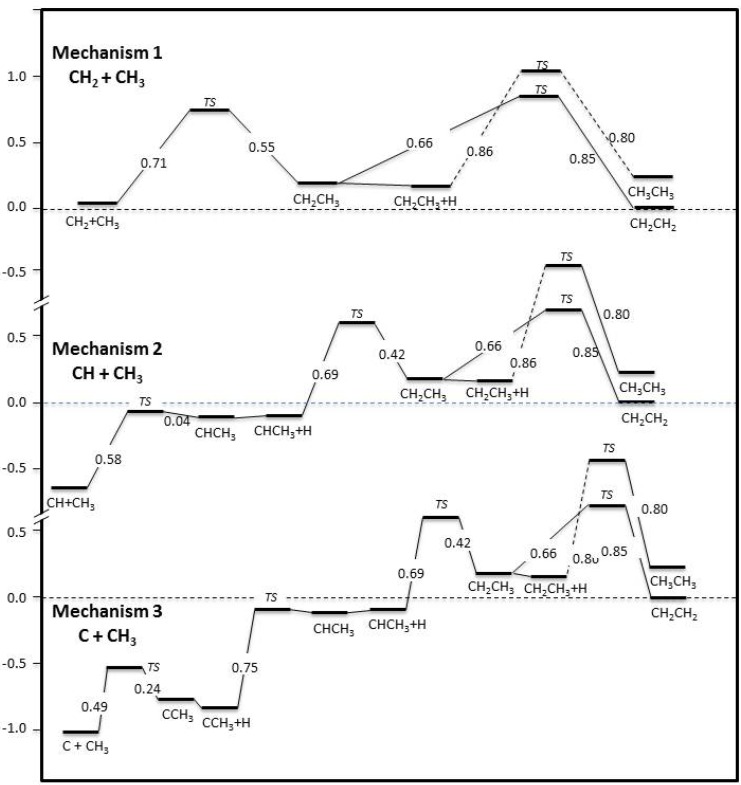
Detailed chemical potential profile characterising the three selected reaction mechanisms. The *y*-axis indicates energy in electronvolts (eV).

Three different pathways towards ethyl formation on Fe(100) have been identified in Figure 3 as most favourable, including CH_2_+CH_3_, CH+CH_3_ (followed by one hydrogenation) and C+CH_3_ (followed by two subsequent hydrogenations), with barriers of 0.71 eV, 0.58 eV and 0.49 eV, respectively. All other couplings of CH_x_ + CH_y_ possess barriers that are substantially higher, and are unlikely. The selected C-C coupling steps have similar barrier values; therefore the parallel operation of all three is to be expected, rather than a single dominant mechanism. Figure 3 shows the details of the *three* selected mechanisms, including C-C step, and subsequent hydrogenations (when necessary) towards ethylene and ethane, via ethyl formation. 

In Mechanism 1, co-adsorbed methyl and methylene would require 0.71 eV in order to couple and form adsorbed ethyl. Ethyl would then need to cross a 0.86 eV barrier in order to further hydrogenate and form ethane, or it could dissociate to ethylene, for which a lower activation energy, 0.66 eV, is required. Ethylene is thermodynamically more stable on Fe(100) and its formation from ethyl is actually exothermic, rather than the less favourable endothermic reaction to form ethane. Note that Mechanism 1 is in essence the one proposed by Biloen and Sachtler [13], which is often quoted in Fischer-Tropsch literature. 

Co-adsorbed methylidyne and methyl, in Mechanism 2, would cross a barrier of 0.58 eV in order to couple and form ethylidene, which happens to be a reversible reaction. In fact, the C-C dissociation reaction of ethylidene has a low barrier of 0.04 eV. In case the forward C-C coupling reaction proceeds, ethylidene will be hydrogenated to form ethyl, a process that requires an activation energy of 0.69 eV. Ethylene and ethane formation would proceed as previously explained.

Co-adsorbed atomic carbon and methyl on Fe(100) would require climbing a barrier of 0.49 eV in order to couple as per Mechanism 3. Two subsequent hydrogenations occur with similar activation energies to those of the methanation reaction on Fe(100) [58], viz 0.75 eV to ethylidene and 0.69 eV to ethyl. The barrier for C-H dissociation of ethylidene is 0.02 eV, very similar to that for its C-C dissociation, as seen in Mechanism 2.

The *total* and *apparent forward barriers* for ethane and ethylene formation, respectively, forMechanism 1 are 1.02 eV and 0.82 eV; for Mechanism 2, 1.67 eV and 1.47 eV; and for Mechanism 3, 2.08 eV and 1.88 eV. Hence Mechanism 3 has an overall barrier which is probably not compatible with the 625 K maximum reaction temperature of the high-temperature Fischer-Tropsch reaction. Both stepwise and in total, the formation reactions for ethylene and ethane–as well as for methane [58] are endothermic, making Fe(100) particularly reactive towards decomposition.

### 2.6. Co-Existence of Many Surface Species: An Interconversion Scheme

**Figure 4:**
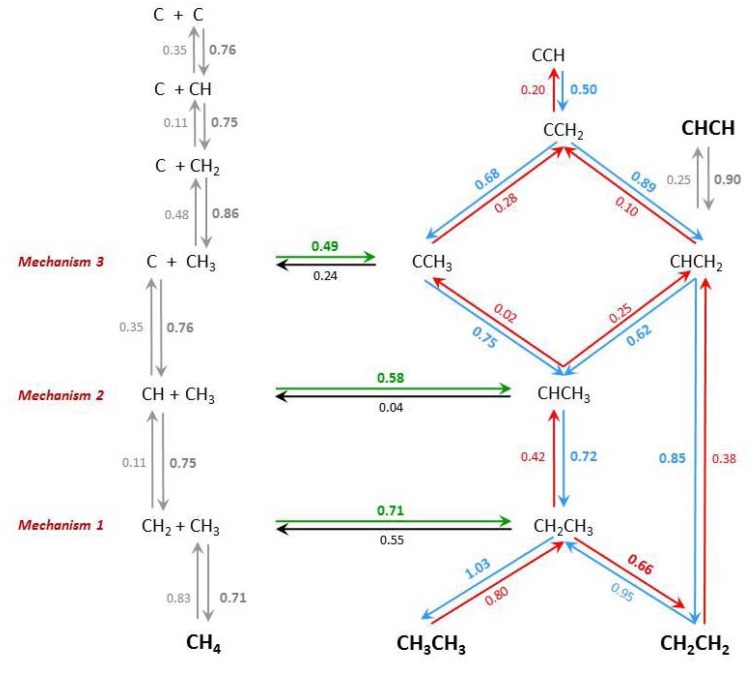
Comprehensive diagram describing a selection of surface reactions of C_2_H_x_ species on Fe(100); the selection considers barriers below 1 eV for C-C bond formation reactions.

The selected mechanisms exhibit substantial overlap with each other; therefore, merging all three into a single scheme would lead to a compact reactivity description of the C_2_H_x_ series. Figure 4 shows a comprehensive scheme with selected reaction steps that lead to the formation of the hydrocarbon series C_2_H_x_ (x = 0–6), and includes both forward and reverse energy barriers (useful when considering competing reactions). Note that we do not claim to be complete, we left out steps with high barriers, or steps for which favorable competing steps are available. Moreover, the selection has been carried out with the formation reaction in mind, rather than the decomposition of species, although several of these have been included. We realise that the present analysis is based on energy barriers only, and we acknowledge that entropic effects may exert a profound role in the rate constants of the elementary steps that we studied. In addition, distributions of reactant species participating in the reaction step as well as their mobility are factors that are expected to have profound influence. We nevertheless hope that the present analysis may contribute to insight in the complexity of Fischer-Tropsch mechanisms. 

Figure 4 illustrates that it would be hard to decide on a ‘most abundant surface intermediate’ during C_2_H_x_ formation on Fe(100) (microkinetic simulations may provide further insight). On the other hand, the prominent role of ethyl, C_2_H_5_, as a precursor to both C_2_H_4_ and C_2_H_6_ stands out in Figure 4, although a competing path to ethylene via vinyl appears feasible, albeit with a somewhat higher barrier. Note also that acetylene, not a common product in Fischer-Tropsch synthesis, cannot be formed directly, but may result by indirect reaction and dehydrogenation of species richer in hydrogen. 

## 3. Computational Method

Projector augmented-wave (PAW) [63,64,65] total-energy calculations, geometry optimisations with analytic forces, normal mode analysis from finite-difference force-constant computation and diagonalisation of the dynamical matrix; and *climbing-image nudged-elastic-band* [66] minimum-energy path computations are carried out by means of the Vienna *ab Initio* Simulation Package (VASP, version 4.6.31).

A kinetic energy cut-off of 400 eV is used for the plane-wave expansion of the single-particle wave-function. The Perdew-Wang 91 (PW91) [67] exchange and correlation energy-functional and potential of *spin-density functional theory* is employed for the self-consistent RMM-DIIS electronic cycles and for building the provided atomic PAW potentials for C, H, O and Fe. Fractional occupancies of the bands is ensured by the first-order Methfessel-Paxton [68] approach, with a 0.1 eV broadening parameter. The RMM-DIIS method is employed for geometry optimisations, as well as for the electronic cycles.

The Fe(100) surface model uses the slab method with a p(2x2) two-dimensional supercell and four metal layers, as well as a vacuum size equivalent to six metal layers. Adsorbates and co-adsorbates sit on one side of the slab, with the top metal layer and adsorbed species allowed to relax. A 5x5x1 Monkhorst-Pack *k*-point sampling [69] is utilised for the two-dimensional Brillouin-zone integrations of surface calculation. A convergence criterium of 0.01 eV/Å is used for the geometry optimisations, normal mode analysis and minimum-energy path computations. The resulting PW91 *bcc*-Fe bulk lattice constant is 2.8313 Å, to be compared with the experimental value of 2.8665 Å. The resulting *spin-only magnetic moment* for *bcc*-iron is 2.16 μ_B_, which is in fair agreement with the experimental value, 2.22 μ_B_.

*Zero-point energy* corrections, as described by Govender *et al.* [58], have been applied throughout. All adsorbate and co-adsorbate stable systems have been characterised as energy minima when all normal modes have real frequency values. Transition states are first order saddle points which contain just one imaginary frequency.

## 4. Conclusions

The formation of C_2_ hydrocarbons, starting from adsorbed atomic hydrogen, carbon and hydrogenated C_1_ adsorbates on Fe(100), has been described as sequentially endothermic as a function of adsorbate complexity, viz as a new hydrogen or a new carbon is sequentially added. Adsorbed ethyl plays the role of main precursor towards the formation of ethylene and ethane, the products of our model reaction.

A systematic first-principles investigation of elementary reaction steps lead to the build-up of a comprehensive scheme comprising three independent mechanisms, nominally methyl and methylene direct coupling, methyl and methylidyne direct coupling followed by one hydrogenation and methyl and adsorbed carbon direct coupling followed by two hydrogenations. All of these mechanisms have been merged into a single one showing the complexity of unraveling a (simplified) reaction mechanism for even the smallest molecules in the FT reaction. Our results seem to indicate that traditional mechanisms of the FT reaction as described in books and papers are definitely oversimplified.

The fact that all pathways towards methane, ethylene and ethane are endothermic illustrates how the iron (100) surface is too reactive towards the adsorbed hydrocarbon species, such that decomposition of the molecules is favored. Of course, in practice, iron catalysts convert into iron carbides, with a surface of reduced activity compared to that of the metal. In this respect, the present study cannot claim to represent a model for the Fischer-Tropsch active surface, and only presents a reference case for the initial stages of the synthesis with a freshly reduced catalyst. 
